# Rhabdomyosarcoma Arising in a Giant Congenital Melanocytic Nevus: A Rare Entity

**DOI:** 10.1007/s13193-025-02457-8

**Published:** 2025-11-01

**Authors:** Samreen S. Qureshi, Parag Ingle, Poonam Panjwani, Vasundhara Smriti, Sajid S. Qureshi

**Affiliations:** 1 Division of Paediatric Surgical Oncology, Department of Surgical Oncology, Advanced Centre for Training Research and Education in Cancer (ACTREC), Tata Memorial Hospital, Tata Memorial Centre, Ernest Borges Road, Parel. 400012, Mumbai, India; 2 Homi Bhabha National Institute (HBNI), Mumbai, India; 3 Department of Pathology, Advanced Centre for Training Research and Education in Cancer (ACTREC), Tata Memorial Hospital, Tata Memorial Centre, Mumbai, India; 4 Department of Radiodiagnosis, Advanced Centre for Training Research and Education in Cancer (ACTREC), Tata Memorial Hospital, Tata Memorial Centre, Mumbai, India

**Keywords:** Rhabdomyosarcoma, Congenital melanocytic nevus, Malignant melanoma, Surgery, Chemotherapy

A 2-year-old boy presented with swelling on his back that had persisted for 4 months, prompting a diagnostic biopsy at a primary care facility. The child was found to have a giant congenital melanocytic nevus with residual swelling in the area (see Fig. 1A). Although a clinical diagnosis of malignant melanoma was initially suspected, the biopsy results revealed an embryonal rhabdomyosarcoma (see Fig. 2). To further evaluate the condition, a positron emission tomography (PET) scan was performed. This scan identified an isolated, fluorodeoxyglucose-avid mass on the back, spanning from the D5 to D12 vertebrae, measuring 7.2 × 2.5 × 8.3 cm (see Fig. 3). Risk stratification categorized the disease as intermediate risk (stage III, Group III, embryonal histology). The patient was treated with induction chemotherapy according to the Intergroup Rhabdomyosarcoma Study (IRS) IV protocol, which resulted in a partial response (see Fig. 1B). Subsequently, a wide local excision was performed, including the previous surgical scar. Primary closure was possible after achieving clear surgical margins. Following surgery, the patient underwent postoperative radiotherapy (41 Gy) and maintenance chemotherapy.

**Fig. 1:**
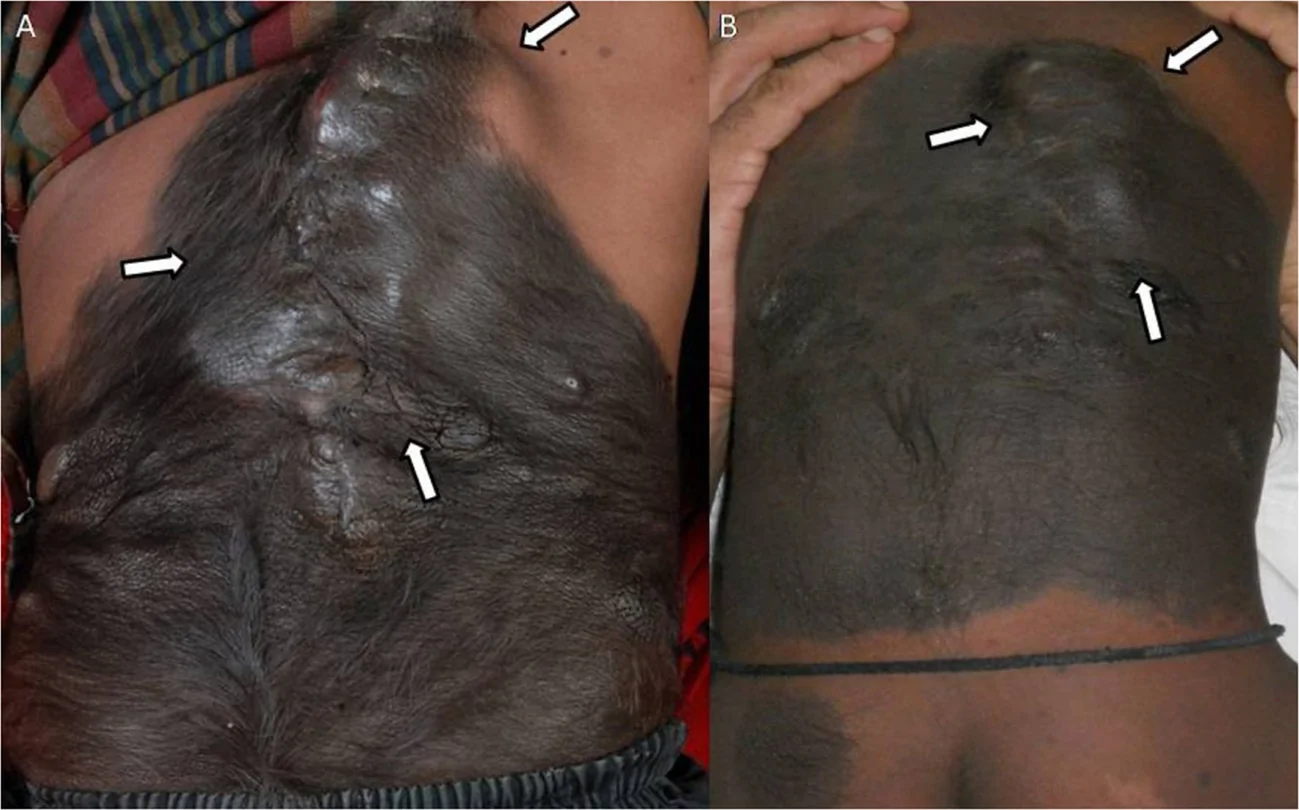
Serial clinical images showing the large congenital hairy nevus in the back with the underlying mass at presentation (**A**) and after induction chemotherapy (**B)**

**Fig. 2:**
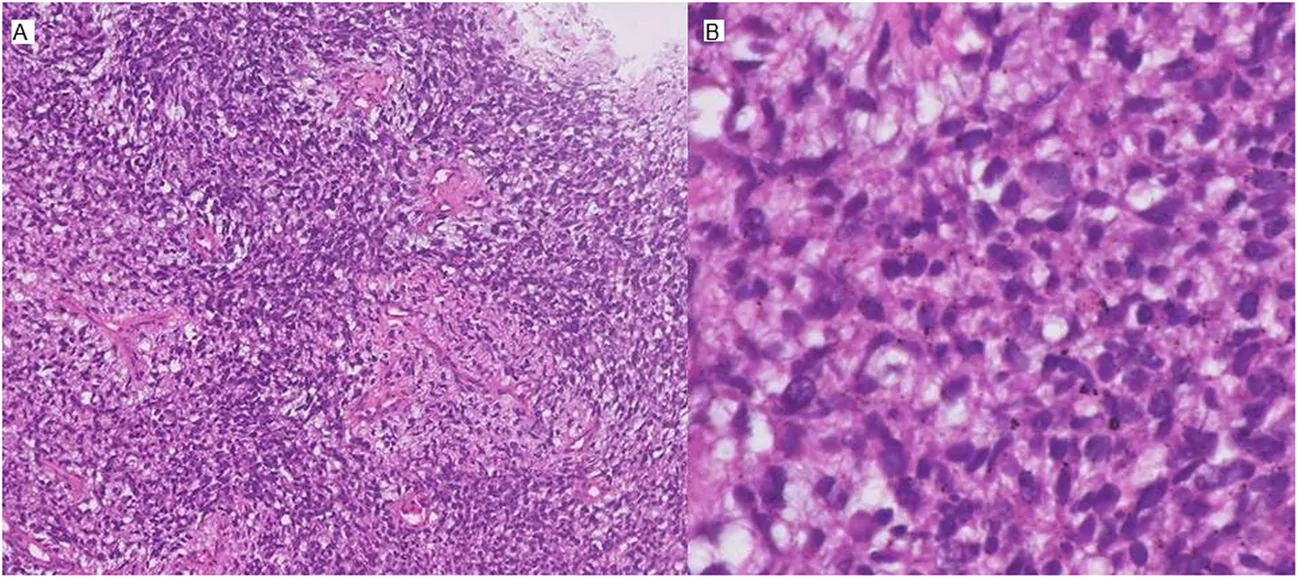
Microphotograph showing cellular spindle cell tumour in short interlacing fascicles (**A**) and high N:C ratio (**B**). (Hematoxylin and eosin, magnification ×10 and ×40)

**Fig. 3:**
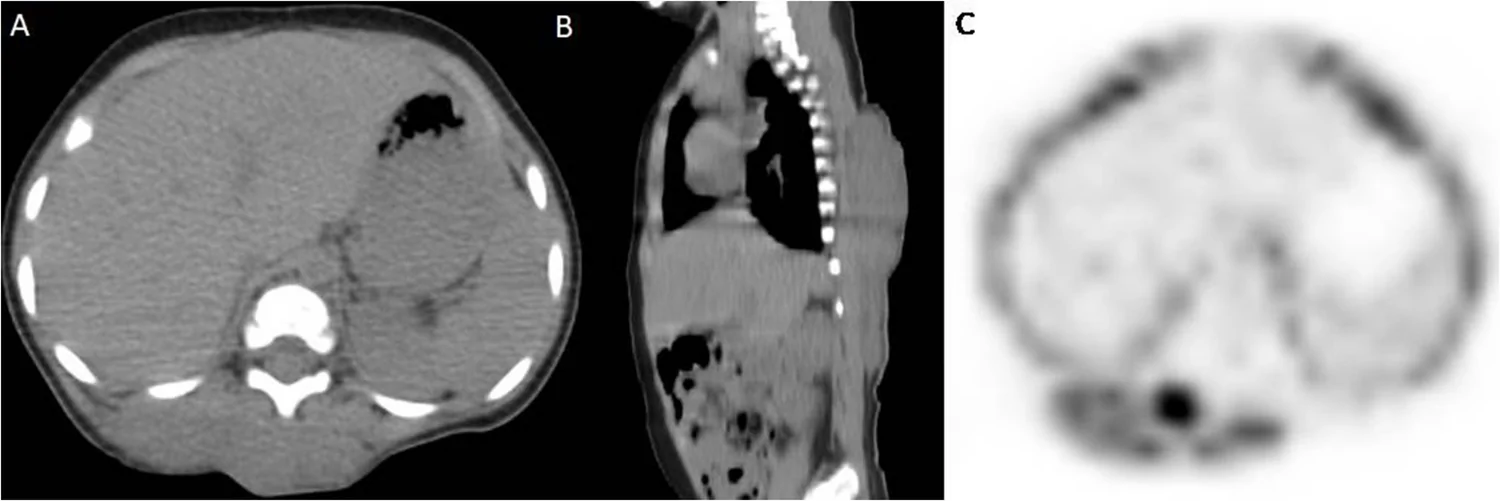
Non-contrast axial (**A**) and sagittal (**B**) computed tomogram component of positron emission tomography (PET) image shows a lobulated soft tissue mass in the back crossing the midline and involving the paraspinous muscles. It shows uptake on the PET image (**C**)

A PET scan performed at the end of the treatment showed no evidence of disease at the primary site or elsewhere. The patient is on regular follow-up with clinical examinations and was enrolled in the institutional After Completion of Treatment (ACT) clinic after 3 years of being disease-free to monitor growth and treatment complications. Local imaging was conducted during enrolment, and the patient is now monitored with clinical examinations, with radiological assessments reserved for instances where symptoms arise.

At the 6-year follow-up, the child remained disease-free with a stable nevus. The occurrence of melanoma in the congenital melanocytic nevi population is significantly high as compared with the general population, with an incidence of 237.56 per 100,000 person-years in the congenital melanocytic nevi population [1, 2]. While malignant melanoma is the most common malignancy associated with congenital melanocytic nevi, the occurrence of rhabdomyosarcoma in this context is extremely rare [3, 4]. Thus, it is crucial to maintain a high level of suspicion in these patients, as the treatment and prognosis differ significantly from those for malignant melanoma.

## Data Availability

Data supporting this study is included within the article.
